# Development and Evaluation of Quality Metrics for Bioinformatics Analysis of Viral Insertion Site Data Generated Using High Throughput Sequencing

**DOI:** 10.3390/biomedicines2020195

**Published:** 2014-05-06

**Authors:** Hongyu Gao, Troy Hawkins, Aparna Jasti, Yu-Hsiang Chen, Keithanne Mockaitis, Mary Dinauer, Kenneth Cornetta

**Affiliations:** 1 Department of Medical and Molecular Genetics, Indiana University School of Medicine, IB 130, 975 West Walnut Street, Indianapolis, IN 46202, USA; E-Mails: hongao@iu.edu (H.G.); hawkinst@gmail.com (T.H.); ajasti@iupui.edu (A.J.); 2 Department of Biology, Indiana University–Purdue University, Indianapolis, IN 46202, USA; E-Mail: yuhschen@iupui.edu; 3 Department of Biology and Center for Genomics and Bioinformatics, Indiana University, Bloomington, IN 47405-3700, USA; E-Mail: kmockait@indiana.edu; 4 Department of Pediatrics and Pathology and Immunology, Washington University, St. Louis, MO 63110, USA; E-Mail: dinauer_m@kids.wustl.edu; 5 Departments of Medicine, Indiana University School of Medicine, Indianapolis, IN 46202, USA; 6 Microbiology and Immunology, Indiana University School of Medicine, Indianapolis, IN 46202, USA

**Keywords:** viral insertion site, quality metrics, next-generating sequencing, integration site PCR

## Abstract

Integration of viral vectors into a host genome is associated with insertional mutagenesis and subjects in clinical gene therapy trials must be monitored for this adverse event. Several PCR based methods such as ligase-mediated (LM) PCR, linear-amplification-mediated (LAM) PCR and non-restrictive (nr) LAM PCR were developed to identify sites of vector integration. Coupling the power of next-generation sequencing technologies with various PCR approaches will provide a comprehensive and genome-wide profiling of insertion sites and increase throughput. In this bioinformatics study, we aimed to develop and apply quality metrics to viral insertion data obtained using next-generation sequencing. We developed five simple metrics for assessing next-generation sequencing data from different PCR products and showed how the metrics can be used to objectively compare runs performed with the same methodology as well as data generated using different PCR techniques. The results will help researchers troubleshoot complex methodologies, understand the quality of sequencing data, and provide a starting point for developing standardization of vector insertion site data analysis.

## 1. Introduction

Gene therapy, using retroviral (RV) and lentiviral (LV) vectors, holds great promise for treatment of a wide variety of genetic disorders and diseases. These integrating vectors, however, have the risk of insertional mutagenesis and thus cause unintended consequences when integration occurs in or near host genes involved in regulating cell growth and division [1,2,3,4]. Therefore, accessing the safety of these gene delivery vectors is of significant importance to the gene therapy community. To elucidate patterns of vector integration, researchers have developed several techniques to identify and characterize integration loci within genomic DNA. Ligase-mediated (LM) PCR [5,6,7,8], linear-amplification-mediated (LAM) PCR [9,10,11], and most recently non-restrictive (nr) LAM PCR [12,13] select and amplify regions of genomic DNA immediately flanking terminal vector sequences. When these fragments are sequenced they can be mapped onto a reference genome for further analysis. Integration loci identified by these methods have been a useful tool for understanding the dynamics of gene-corrected hematopoietic stem cells in transplantation models. They have been used as biomarkers for tracking the growth and distribution of individual clones during repopulation [14].

LM, LAM, and nrLAM PCR (subsequently referred to as integration site PCR or IS-PCR) are all variations of a general method for identifying the site of vector integration. Specific primers for the vector Long Terminal Repeat (LTR) are utilized along with adaptor sequences for the adjacent genomic region that permit amplification of sequences at the site of integration. Amplification may also generate a second sequence, a LTR-internal vector sequence, due to the redundancy in the 5' and 3' LTR. The latter sequence serves as an internal control for the amplification reaction. LM- and LAM-PCR rely on restriction enzymes digestion that provide bias and do not detect integration that are distant from the vector integration site; requiring the use of multiple reactions with different enzymes to increase the sensitivity of detection. nrLAM-PCR does not utilize restriction enzymes and therefore can decrease the number of reactions required, but the level of sensitivity of detecting sequences among the three methods has not been carefully compared.

LM-PCR and LAM-PCR amplicons can be separated on electrophoresis gel and individual bands subjected to shot gun cloning and Sanger sequencing. The product of nrLAM-PCR appears as a smear on a high-resolution gel instead of distinct bands and downstream analyses of nrLAM-PCR products are solely dependent on sequencing [12]. Sanger sequencing of IS-PCR products has now been replaced by next-generation sequencing (NGS) methods that are less time intensive and greatly increase the data obtained from complex samples, such as those obtained after transduction of cell populations used in hematopoietic stem cell transplantation and adoptive immunotherapy. Researchers have achieved comprehensive and useful integration site information by coupling LAM-PCR and NGS [15,16,17,18,19]. While NGS technologies provide clear technical advantages, the use of sequence-capture and PCR amplified products, along with potential technical variation in sequencing platforms, could fail to detect an integration site. As these methods are being used to monitor subjects participating in human gene therapy trial for adverse events, understanding the quality of the sequencing data is critical.

In this paper, we define a series of metrics for assessing NGS data derived from IS-PCR products. The goal of the study is not to directly compare the IS-PCR methods; rather we seek to develop metrics that link the quality criteria used for processing NGS data irrespective of the IS-PCR methodology. We will describe the development of five simple metrics that can be universally applied to data generated from IS-PCR and NGS and show how the metrics can be used to objectively compare data, using LM, LAM and nrLAM-PCR data for illustration. In addition to providing standardization of a specific IS-PCR method, the quality metrics will have utility in providing researchers with a rubric for troubleshooting complex methodologies, understanding the resulting data more comprehensively, and provide a basic framework for evaluating novel IS-PCR technologies.

## 2. Results and Discussion

In this study we developed a series of quality metrics and applied these to sequence data obtained using next generation sequencing technology. The analysis includes a comparison of LM, LAM, and nrLAM-PCR techniques using K562 clones with known integration sites for a HIV-1-based lentiviral vector expressing eGFP. The clones, designated as clone 3 and clone 6, have 2 integrations per cell. The specificity and sensitivity of the different IS-PCR methods were assessed by analyzing 31 samples that contained various percentage of genomic DNA from the two clones (Table 1).

**Table 1:** Lentiviral vector transduced K562 clones used in the study.

Clones	Ratio	Protocol
K562 clone 3 + K562 clone 6	100:0	LM, LAM, nrLAM
K562 clone 3 + K562 clone 6	99.9:0.1	LM, LAM, nrLAM
K562 clone 3 + K562 clone 6	99:1	LM, LAM, nrLAM
K562 clone 3 + K562 clone 6	90:10	LM, LAM, nrLAM
K562 clone 3 + K562 clone 6	75:25	LM, LAM, nrLAM
K562 clone 3 + K562 clone 6	50:50	LAM, nrLAM
K562 clone 3 + K562 clone 6	25:75	LAM, nrLAM
K562 clone 3 + K562 clone 6	10:90	LM, LAM, nrLAM
K562 clone 3 + K562 clone 6	1:99	LM, LAM, nrLAM
K562 clone 3 + K562 clone 6	0.1:99.9	LM, LAM, nrLAM
K562 clone 3 + K562 clone 6	0:100	LM, LAM, nrLAM

The PCR products of LM-PCR, LAM-PCR, and nrLAM-PCR reactions were subjected to a further PCR step to incorporate adaptors to generate multiplexed libraries and PCR amplicons from each sample were pooled and subjected to 454 pyrosequencing on Roche 454 FLX Titanium sequencer. We obtained a total of 56,622 raw reads from 9 LM-PCR samples, 72,121 raw reads from 11 LAM-PCR samples, and 25,807 raw reads from 22 nrLAM-PCR samples (sequenced in duplicates).

### 2.1. Theoretical Yield

**Figure 1:**
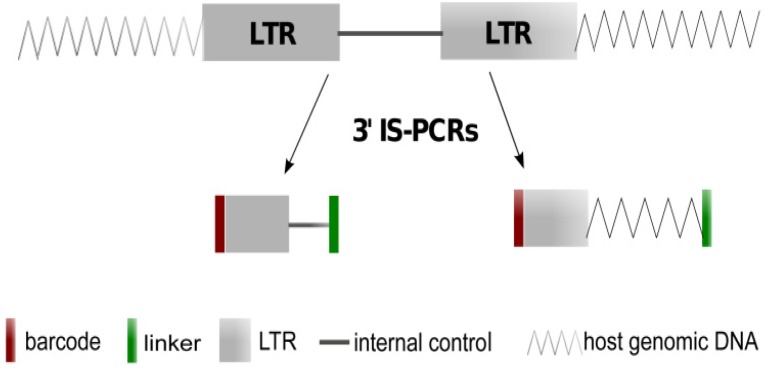
Features used for developing metrics. The figure illustrates integration of vector into host genomic DNA. In this representation the target initiation sequence for integration site PCR (IS-PCR) are in both Long Terminal Repeats (LTRs) thus generating products that extend into the 3' genome and into the vector (internal control).

**Figure 2:**
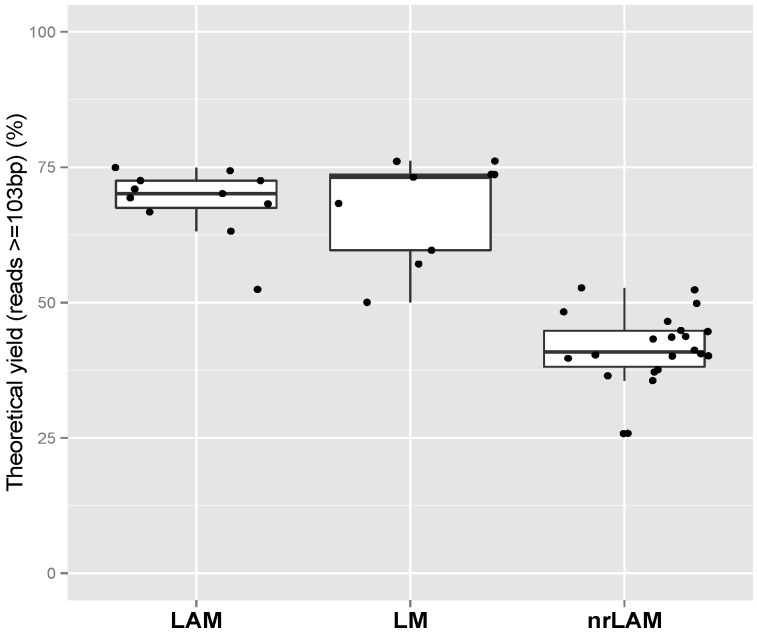
Comparison of Theoretical Yield (TY) values between different IS-PCR approaches.

LM, LAM, and nrLAM PCR are all based on the same principle of capture and amplification of junctions between vector LTR and genomic DNA; the basic structure of the final product of all three methods is the same. We expect each sequence read to contain a bar code and a known portion of the vector LTR with either genomic or internal vector sequence (Figure 1). We define the Theoretical Yield (TY) as the percentage of reads of sufficient size to be potentially informative in identifying the site of integration. For our vector, we determined that the primers utilizes in IS-PCR would generate 83 bp of LTR. Also, analysis by our laboratory and others [12] require 20 bp of flanking sequence to be included in the read to reliably identify a unique integration. Therefore, sequences of 103 bp or greater were considered potentially informative after the reads were processed to remove the linker and adaptor sequences. The TY represents the number of reads of 103 bp or greater divided by the total number of reads. Figure 2 showed the distribution of TY for LM-PCR, LAM-PCR and nrLAM-PCR. The average TY values for LM-PCR, LAM-PCR and nrLAM-PCR are 67.54%, 68.67% and 41.38%, respectively. LM-PCR and LAM-PCR have comparable TY values (*p*-value = 0.71, alpha = 0.05) whereas nrLAM-PCR has the lowest TY value which is significant different from that of LM-/LAM-PCR (*p*-value < 4 × 10^−7^, alpha = 0.05). The lower TY value of nrLAM-PCR is not unexpected as nrLAM-PCR produces amplicons of various lengths because there is no restriction enzyme digestion in the procedure; shorter products could be preferentially amplified in the exponential amplification step increasing the relative number of sequence less that 103 bp.

When applying these quality metrics, each laboratory can define a range of expected TY values specific to their methodology and vector characteristics. When a sample falls outside the expected range, the sample should be considered for further scrutiny. For example, LM- and LAM-PCR products can be further evaluated on agarose gels; a poor TY with defined bands on the gel would suggest difficulties with the sequencing reaction or immediately preceding preparation steps. Decision can then be made whether the IS-PCR reaction and sequencing needs to be repeated or whether repeat sequencing alone is required.

### 2.2. Vector Specificity

**Figure 3:**
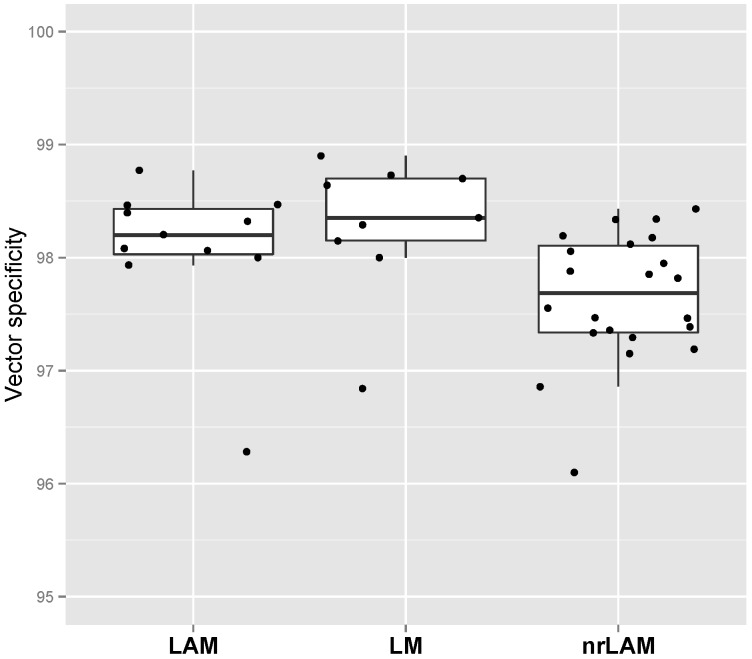
Comparison of vector specificity (*S*_v_) between different IS-PCR approaches.

The next quality measure evaluated the percentage of reads that are 103 nucleotides in length or greater that contained the LTR sequence, a quality measure of capture efficiency we refer to as vector specificity (*S*_V_). Since IS-PCRs utilize sequence capture and PCR to enrich sequences adjacent to the inserted LTR, an efficient reaction would produce products in which the majority of read contain LTR sequences. Therefore, *S*_V_ is a measure of the quality of the IS-PCR reaction. We observed that greater than 97% of all reads (relative to TY) obtained in our study carried vector LTR sequence, with average *S*_V_ values of 98.29%, 98.09%, 97.65% for LM-PCR, LAM-PCR and nrLAM-PCR, respectively (Figure 3). There was no significant difference between the methodologies confirming the published methods we utilized for IS-PCR provide a high rate of sequence capture. If a sample were to have a low *S*_V_ troubleshooting can be focused on non-specific primer binding or inefficient capture of LTR-containing amplicons. Also, when developing primers and conditions for new vector systems, an evaluation of *S*_V_ can provide assurance that the new methodology provides comparable reaction characteristics.

### 2.3. Internal Control and Genomic Specificity

**Figure 4:**
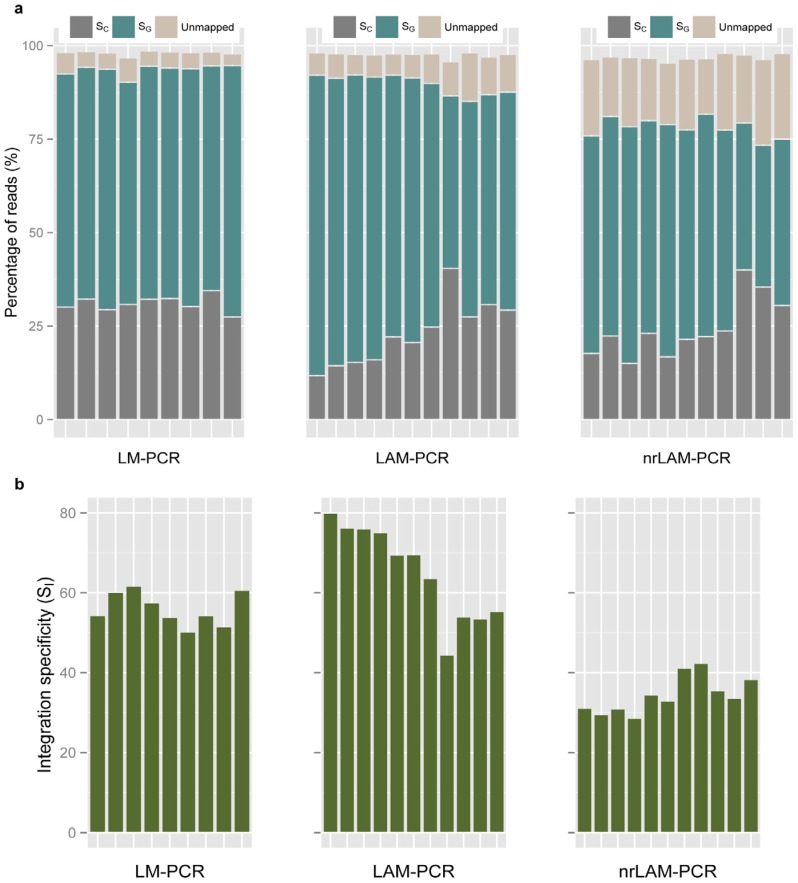
Comparison of Internal control specificity (*S*_C_), genomic specificity (*S*_G_) and integration specificity (*S*_I_) between different IS-PCR approaches. (**a**) *S*_C_, *S*_G_ and percentage of unmapped reads; (**b**) *S*_I_ between different IS-PCR methods. Each bar represented a sample.

The third quality metric evaluated is the fraction of reads that contain the internal vector sequence and are equal or greater than 103 bp, a measure we called the internal control specificity (*S*_C_). In theory, half of the reads of appropriate length should represent the internal control product because the primers used in IS-PCR have homology in both the 5' and 3' LTR. Our experiment resulted in an average *S*_C_ value of 31.01%, 22.97% and 24.37% for LM-CPR, LAM-PCR and nrLAM-PCR (Figure 4a). The *S*_C_ value for LAM-PCR is the lowest among the three. In our study, we utilized the same enzyme in the LM-PCR and LAM-PCR (*Tsp*509I) and both are predicted to generate an internal control band of 210 bp. It may be that other difference in the reaction led to differential amplification of the internal control band. For example, during the linker ligation LAM uses sticky-end ligation while LM-PCR uses blunt end ligation. Also, the LM-PCR enzyme digestion is performed before PCR extension. The low *S*_C_ value for LAM-PCR could occur if a significant number of the internal control amplicons did not reach the first *Tsp*509I recognition site of the vector, which prevented the addition of the linker sequence and therefore resulted in poor yield of final internal control product. The use of an internal control is not required and not all investigators chose restriction enzymes within the vector genome. Therefore, assessments of quality can be performed without *S*_C_ but if an internal control band is generated this metric allows one to evaluate consistency between samples and set expectations when altering existing IS-PCR protocols.

**Figure 5:**
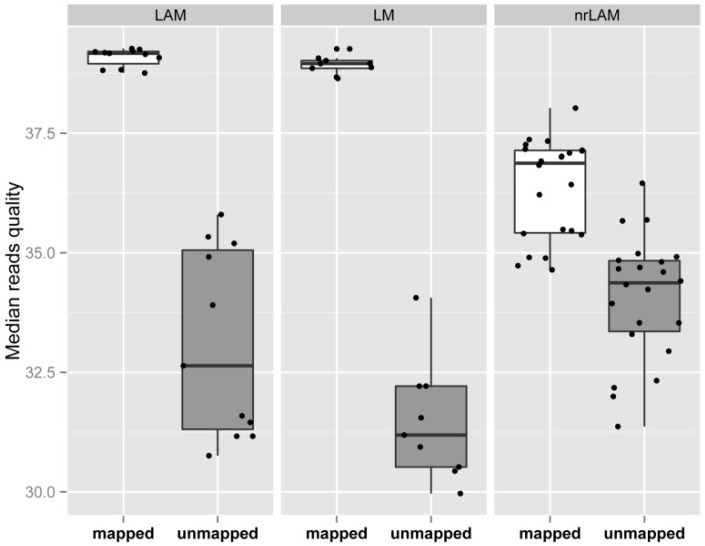
Read quality of mapped and unmapped reads. Median read quality (Phred quality) was calculated for each read in a sample. The median qualities of all mapped and unmapped reads in a sample were averaged for comparison.

The fourth quality metric, the genomic specificity (*S*_G_), describes the fraction of sequence reads that map to one or more locations in a reference genome. As described in Methods, sequencing read are first processed by removing portions of reads aligning to bar code, LTR and adaptor, and internal vector sequences were removed before mapping reads to a reference genome. We found that the average *S*_G_ for LM-, LAM- and nrLAM-PCR were 62.56%, 66.73% and 53.66%, respectively (Figure 4a). The metrics *S*_C_ and *S*_G_ can be combined (*S*_C_ + *S*_G_) to represent the fraction of total reads that successfully map to expected sequences. Therefore, the sum of informative reads (*i.e*., that map to the reference genome) and reads that containing the internal vector sequence represent another assessment of quality. In our evaluation, *S*_C_ + *S*_G_ was 92.57%, 89.70% and 78.03% for LM-, LAM-, and nrLAM-PCR, respectively. In our analysis it was also informative to evaluate the unmapped reads; unmapped reads had an overall lower read quality (*p*-value < 0.001 for all three IS-PCR methods) (Figure 5).

### 2.4. Integration Specificity

Integration specificity (*S*_I_) measures the reads that mapped uniquely to the reference genome, which is predicted to be the precise site of vector integration. This differs from the metric *S*_G_ which measures the percentage of reads that mapped to one or more regions. This distinction is important given the large number of repeat regions in the genome. We therefore sought to identify *S*_I_ by requiring that the IS-PCR sequence mapped to a unique sequence in the reference genome and the end of LTR was no more than 2 bp from the genomic alignment. Comparing the three methods, the average *S*_I_ values were 56.05%, 65.22% and 34.43% for LM-, LAM- and nrLAM-PCR, respectively (Figure 4b). The lower value for nrLAM-PCR is not unexpected; LM- and LAM-PCR generated specific bands while nrLAM-PCR generates a smear when analyzed by gel electrophoresis, *i.e.*, the nrLAM-PCR bands are of variable length many of which are smaller thereby increasing the chance for matching at multiple regions in the genome.

### 2.5. Assaying Lower Limit Sensitivity of the IS-PCR Methods

**Figure 6:**
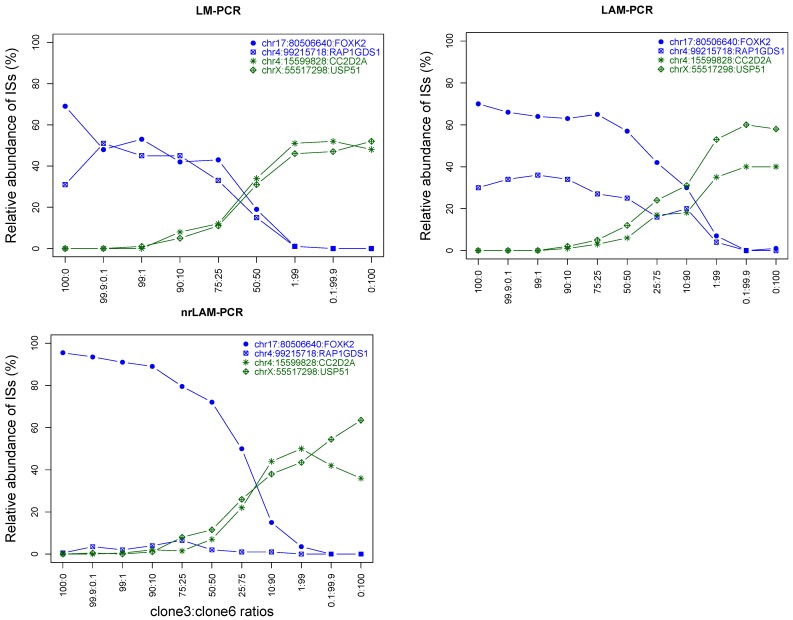
Specificity and sensitivity of different IS-PCR methods. The blue lines represent clone 3 and the green lines represent clone 6. The insertion sites were labeled in the format as chromosome:position:closest gene.

The quality measures described above provide assessment of the sequences returned after HTS but they do not provide assurance that a sequence will be detected. Currently, there is limited data directly comparing the three IS-PCR methodologies evaluated here. Therefore, in addition to the specificity metrics described above, we also directly evaluate the sensitivity at which the different capture and amplification methods could retrieve known integration events. As shown in Table 1, DNA from K562 clones with known integrations were mixed at concentrations ranging from 99.9% to 0.1% and subjected DNA mixtures to IS-PCR followed by 454 pyrosequencing (Figure 6). To estimate the original ratio from the sequencing data, we used a normalized fraction of reads mapping to each known locus.

We found all three IS-PCR methods were able to quantitatively detect DNA from the original mixture (Figure 6). LM-PCR has the highest accuracy in quantitatively tracking the contribution of individual clones in a clonal mixture with an *r*^2^ of 0.997. LAM-PCR and nrLAM-PCR are less accurate in representing the relative abundance of individual clones, where LAM-PCR has an *r*^2^ of 0.896 and nrLAM-PCR of 0.905. The level of sensitivity of detecting a specific integration within a population varied. All three approaches could consistently detect known integration sites when 10% or more of the cell contained an insertion; many but not all insertions were detected at the 1% level (Figure 6). Preliminary results with the MiSeq platform, which provides a greater number of reads, does appear to increase the level of detection more consistently to the 1% range (data not shown). The sensitivity testing was performed when there was an excess of competing insertion sites, it is possible that sensitivity may be higher when analyzing vector containing cells is a population of vector negative cells.

### 2.6. Application of Various Metrics Developed

**Figure 7:**
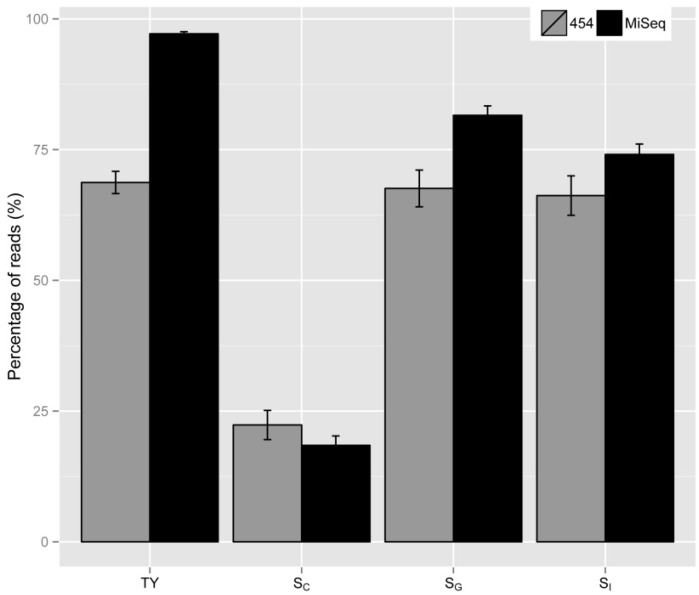
Comparion of various metrics developed on different sequencing platforms.

With the aim to apply the metrics developed on different sequencing platforms, we carried out sequencing on MiSeq using the LAM-PCR products previously used for 454 sequencing. The resulting MiSeq reads were analyzed similar to the 454 reads and various metrics values were calculated. We found the TY values were significantly increased when we included a short (<100 bp) amplicon removal step before library construction (*p*-value = 1.803 × 10^−^^5^, Figure 7). *S*c values were similar (*p*-value = 0.393). The increase of mapped and uniquely mapped sequence values (*S*_G_ and *S*_I_, respectively) was modest (with *p*-values 0.0015 and 0.089 for *S*_G_ and *S*_I_, respectively). This is expected given both the numerators and denominators of *S*_G_ and *S*_I_ were increased.

The quality metrics were also evaluated on more complex samples. We analyzed data derived from X-linked chronic granulomatous disease (CGD) mice transduced with lentiviral vector expressing gp91 phox. LM-PCR and 454 pyrosequencing were carried out on these murine samples as explained in the Material and Methods. The number of insertion sites and various metrics were summarized in Table 2. The TY and *S*_V_ values of these samples are similar to that observed in our K562 clone samples (Figure 2). The *S*_C_ values were higher in these samples than the K562 clones and can be explained by stronger amplification and capture of the internal control bands; an explanation supported by electrophoresis and visualization of the LM-PCR product (data not shown). The *S*_G_ and *S*_I_ values cannot be directly compared with that of the K562 clones because the reference genomes are different (*i.e*., cells of murine *versus* human origin).

**Table 2:** Number of insertion sites and various metrics numbers in transduced murine samples.

Samples ^#^	Number of Insertion Sites *	TY	*S*_V_	*S*_C_	*S*_G_	*S*_I_
Sample 1 bone marrow	57	70.5 ± 0.68	97.73 ± 0.44	44.82 ± 0.96	52.49 ± 0.69	47.25 ± 0.26
Sample 1 spleen	58	64.25 ± 0.57	99.27 ± 0.04	47.32 ± 1.11	50.36 ± 1.06	47.39 ± 0.91
Sample 2 bone marrow	7	79.6 ± 0.32	98.72 ± 0.17	57.75 ± 1.11	40.65 ± 1.03	39 ± 1.03
Sample 2 spleen	24	73.87 ± 0.05	99.22 ± 0.14	50.67 ± 0.24	48.08 ± 0.15	46.74 ± 0.18
Sample 3 bone marrow	33	54.9 ± 0.56	93.54 ± 0.21	49.5 ± 1.19	45.34 ± 1.24	33.1 ± 1.14
Sample 3 spleen	53	65.72 ± 0.71	94.67 ± 0.38	49.69 ± 0.93	44.75 ± 1.04	36.06 ± 0.83
Sample 4 bone marrow	28	67.72 ± 0.68	99.49 ± 0.11	55.48 ± 0.84	42.92 ± 0.65	39.19 ± 0.82
Sample 4 spleen	37	68.83 ± 0.84	99.23 ± 0.12	61.43 ± 0.23	36.68 ± 0.13	32.44 ± 0.33

# Each sample was sequenced three times; * The number of insertion sites identified in all three replicated sequencings.

Several tools have been developed to characterize viral vector integration sites using next generation sequencing technology [20,21,22,23,24]. However, none of these tools tried to establish quality metrics that can be used to assess the data quality derived from different PCR techniques and sequencing platforms. Therefore, the goal of this study is to establish a set of metrics not to determine which IS-PCR is “better”. Each IS-PCR method has advantages and disadvantages that vary with the vector design, frequency of vector copies within a population, and the number of samples requiring analysis. We propose the metrics as a means to assess a particular experiment, to evaluate modifications to an established method, or in method development. For example, we hoped to replace restriction enzyme digestions with hydroshearing. Prior to PCR amplification and sequence capture, DNA was sheared to an average length of 400 bp. Unfortunately, the metrics were significantly poorer than other IS-PCR and identified that further protocols modifications will be required before this method could replace existing techniques.

In practice, values for theoretical yield (TY), vector specificity (*S*_V_), genomic specificity (*S*_G_), and integration specificity (*S*_I_) are expected to be consistent for the particular method utilized. As the measurements are dependent on a good TY value, quality metric values falling outside an expected range would lead to investigation of the PCR conditions. Vector specificity (*S*_V_) is used to validate *bona fide* integration and low values suggest that the primers and PCR protocol could be optimized to improve primer binding or/and amplicon capture. In general, TY and *S*_V_ are metrics that assess the IS-PCR conditions while *S*_G_ and *S*_I_ values are more impacted by the computational tools used to map reads to the reference genome, including the completeness of the target genome. If a sample has appropriate TY and *S*_V_ values, low *S*_G_ or *S*_I_ values may indicate integrations within repeat regions leading to mapping to multiple locations. If samples are from species with poorly annotated genomes, low *S*_G_ or *S*_I_ values are also expected. The need for quality metrics goes beyond their usefulness in troubleshooting technical problems, they can provide increased confidence in the data generated. A major challenge with IS-PCR is the need to detect vector integrations, the sites of which are unknown. While regulators will ask that investigators validate the assay and demonstrate the level of sensitivity, they will also require future reactions be performed in a manner that is comparable to the validation study. Developing metrics will help demonstrate that an IS-PCR reactions fall within a predefined range of values, thereby meeting quality requirements and providing additional assurance that the IS-PCR reaction and analysis will detect integrations with the expected sensitivity.

## 3. Materials and Methods

### 3.1. Metrics Calculation

The theoretical yield (TY) is a readout of the number of reads you expect might contain useful data for a single LM-PCR sample sequenced in high throughput: TY = *N*_*l*>*n*_/*N*_T_, where *l* is read length and *n* is a threshold length that is defined by the length of the LTR after the final round primer position + the minimal length required for significant statistical alignment of a read to a genome sequence; *N*_*l*>*n*_ is the number of reads surpassing that threshold, and *N*_T_ is the total number of returned reads. The vector specificity (*S*_V_) is a readout of the fraction of the returned reads of appropriate length that contain LTR sequence from the vector: *S*_V_ = *N*_V_/*N*_*l*>*n*_ × 100, where *N*_V_ is the number of reads containing vector LTR sequence. Vector specificity is a subset of theoretical yield. The genomic (*S*_G_) and internal control specificity (*S*_C_) are readouts of the fraction of the returned reads of appropriate length and containing LTR sequence from the vector that map to either a genomic location or the internal vector control: *S*_G_ = *N*_G_/*N*_*l*>*n*_ × 100 and *S*_C_ = *N*_C_/*N*_*l*>*n*_ × 100, where *N*_G_ and *N*_C_ are the number of reads mapping to genomic and internal vector sequence, respectively. Genomic specificity and internal control specificity are both part of theoretical yield. The integration specificity (*S*_I_) is a readout of the fraction of the returned reads of appropriate length that contain LTR sequence from the vector and have a genomic alignment immediately flanking the vector sequence: *S*_I_ = *N*_I_/*N*_*l*>*n*_ × 100, where *N*_I_ is the subset of *N*_G_ where the genomic alignment immediately flanks the identified boundary of vector LTR.

### 3.2. Lentiviral Vector Transduced K562 Clones and Murine Hematopoietic Tissue

K562 cells (human immortalized myelogenous leukemia line) were transduced with lentiviral vector (CSCGW, a HIV-1 based third generation lentiviral vector containing the enhanced green fluorescent protein under a CMV promoter kindly provided by Philip Zoltick) at an MOI of 1 for 4 h in the presence of 8 µg/mL of polybrene at 37 °C. The transduced cells were sorted (FACS sorter name) using the GFP marker and plated in 96 well plates for culture to limiting dilution. Clones were expanded and analyzed by Southern blot analysis with two bands document in clone 3 and 6. The site of integration was identified through NGS and confirmed using LTR/integration site PCR primers. The integration sites are chr17:80506640 and chr4:99215718 for clone 3 and chr4:15599828 and chrX:55517298 for clone 6. The clones are available through the National Gene Vector Biorepository (http://www.NGVBCC.org). For the study of primary cells, marrow from mice deficient for X-linked chronic granulomatous disease was transduced with a third-generation lentiviral vector expressing the *X-CGD* gene then injected into X-CGD mice previously irradiated with 300 cGY according to previously reported procedures [25].

### 3.3. LTR Insertions Site Analysis by LM-PCR, LAM-PCR and nrLAM-PCR

A total of 11 genomic DNA samples were generated by mixing DNA from two of the selected K562 clones (Table 1). To retrieve vector-genome junctions, LM-PCR, LAM-PCR and nrLAM-PCR were performed on these synthetic samples. The general schema for each of these methods are illustrated in Figure 1. The steps to perform LM-PCR, LAM-PCR and nrLAM were published previously [10,13]. Briefly, for LM-PCR, 250 ng genomic DNA was digested with *Tsp*509I (NEB) for 2 h at 65 °C, followed by labeled primer extension with an LTR-specific primer (5'-gaacccactgcttaagcctca-3', IDT) and captured on streptavidin-coated magnetic beads (Dynal M-280, Invitrogen, Carlsbad, CA, USA). A blunt-end adaptor oligonucleotide cassette (5'-gtaatacgactcactatagggcactatagggcacgcgtggt-3', IDT) was ligated to the 3' end of the captured fragments, which were then subjected to nested PCR (Round 1: 5'-agcttgccttgagtgcttca-3' and 5'-gtaatacgactcactatagggc-3'; round 2: 5'-agtagtgtgtgcccgtctgt-3' and 5'-actatagggcacgcgtggt-3'). For LAM-PCR, 250 ng genomic DNA was used as a template for 50 cycles of linear PCR with a labeled LTR-specific primer (same as LM-PCR primer extension above) followed by capture of ssDNA on magnetic beads, double-stranding by Klenow polymerase using hexanucleotide primers (Roche, Indianapolis, IN, USA), and digestion as described for LM-PCR. A sticky-end adaptor cassette 5'-AATTCCTAACTGCTGTGCCACTGAATTCAGATC-3', 3'-GGATTGACGACACGGTGACTTAAGTCTAGAGGGCCCAG-5' was ligated to the 3' end of captured and processed fragments, which were then subjected to nested PCR (Round 1: 5'-agcttgccttgagtgcttca-3' and 5'-gacccgggagatctgaattc-3'; round 2: 5'-agtagtgtgtgcccgtctgt-3' and 5'-agtggcacagcagttagg-3'). The resulting products were visualized by gel electrophoresis. For nrLAM-PCR, linear PCR and bead capture was performed as described for LAM-PCR. A single-stranded adaptor (5'-PCCTAACTGCTGTGCCACTGAATTCAGATCTCCCGGGTddC-3') was ligated O/N at room temperature with T4 RNA ligase (Roche) in a reaction tube packed with PEG 8000 and hexa-amine cobalt chloride (Sigma, St. Louis, MO, USA). Nested PCR was performed on the ligation product as described for LAM-PCR. Resulting products were visualized on a gel to confirm random size distribution.

### 3.4. 454 Library Preparation

To sequence the products from LM-PCR, LAM-PCR, and nrLAM-PCR, individually bar-coded amplicon libraries were generated by a further PCR step using forward fusion primers containing the Roche Titanium 454A adaptor, a unique 10-nt multiplex identifier (MID), and an LTR-specific sequences (5'-cca tct cat ccc tgc gtg tct ccg act cag [nnn nnn nnn n] [1] ag tag tgt gtg ccc gtc tgt-3', IDT) and reverse fusion primers containing the Roche Titanium 454B adaptor and LM-PCR adaptor cassette-specific sequences (5'-cct atc ccc tgt gtg cct tgg cag tct cag act ata ggg cac gcg tgg t-3', IDT). Samples were pooled, purified, and sequenced in triplicate on a Roche 454 FLX Titanium instrument at the Indiana University Center for Genomics and Bioinformatics.

### 3.5. MiSeq Sequencing

The first exponential PCR products generated by LAM-PCR on mixtures of clone 3/clone 6 were applied to a further PCR step using forward primers incorporated with different barcodes. The final PCR products were purified and shorter amplicons (<100 bp) were removed with Ampure beads (Agencourt, Beverly, MA, USA). Samples were then pooled into different libraries after DNA quantification with Qubit. The pooled libraries were further processed into MiSeq sequencing libraries using the Illumina TruSeq library construction kit and sequenced on Illumina MiSeq at the Genomics Core Facility of University of Notre Dame. Pair-end reads of 159 bp were generated.

### 3.6. Data Processing and Integration Loci Identification

454 sequencing reads were binned using the 10-nt MID sequences for each originating sample. CUTADAPT version 1.1 [26], cross_match version 0.990329 (part of Phrap package by Phil Green: http://www.phrap.org) and in-house written scripts were used to remove MID sequences, adaptor sequences, linker sequences, lentiviral LTR sequences and internal control reads from each sample before genomic alignment. To identify integration sites, valid reads carrying 20 bp or more of human genomic sequence right adjacent the LTR sequence were then mapped to the human genome (hg19) using Bowtie [27]. Reads were discarded if mapped to multiple sites. The mapped reads were annotated using the hg19 refGene bed files (UCSC Genome Database). MiSeq reads were processed in a similar way except the pre-processing steps like reads binning and adaptor removal. All the figures were generated in R with the package ggplot2 [28,29].

## 4. Conclusions

In summary, the development of next-generation sequencing (NGS) technologies has provided the capacity to rapidly obtain accurate sequence data. Coupling this technology with various IS-PCR approaches will permit a more comprehensive and genome-wide profiling of insertions sites and increase throughput by several orders of magnitude. Researchers have achieved richer integration site information by applying NGS [12,15,16,17,18,19], but regulators are now expecting this method to be used for monitoring patients for insertional mutagenesis. Moving the method from a research test to one that will be used for clinical decision-making raises the bar in terms of reproducibility and standardization. As different IS-PCR methodologies have their own advantages and disadvantages the choices of IS-PCR for patient monitoring will likely vary between clinical trials and between different laboratories. Nevertheless, the quality metrics described here is an initial step to provide standardization since the metrics can be established of any IS-PCR method. Once a laboratory established the expected range of quality metrics for their particular IS-PCR assay, the metrics can be monitored at various stages in the bioinformatics analysis and provide assurance that the data generated from clinical samples meet pre-defined acceptability criteria. Our initial step towards standardization will be critical if IS-PCR methodologies are to assist in monitoring gene therapy patients for insertional mutagenesis.
